# Establishment and population expansion of the non‐native Azores chromis (*Chromis limbata*) in the subtropical southwestern Atlantic

**DOI:** 10.1111/jfb.70139

**Published:** 2025-07-14

**Authors:** Rafael Romero Munhoz, Ana Clara Sá Athayde, Caio Ribeiro Pimentel, Fernanda Andreoli Rolim, Guilherme Henrique Pereira‐Filho, Fábio dos Santos Motta

**Affiliations:** ^1^ Marine Ecology and Conservation Lab (LABECMar) Federal University of São Paulo São Paulo Brazil

**Keywords:** Brazil, invasive species, MPA, new records, reef fish

## Abstract

This study documents new records and the spatial–temporal progression of the non‐native Azores chromis (*Chromis limbata*) in southeastern Brazil, particularly on the coast of São Paulo state. We report the first confirmed occurrence of *C. limbata* in the Alcatrazes Archipelago and document that, in recent years, its abundance has exceeded that of the native brown chromis (*Azurina multilineata*). These findings indicate potential shifts in the taxonomic composition of reef fish communities and highlights the importance of further research to understand the processes driving these patterns and their ecological implications.

Non‐native species pose a global challenge to biodiversity, ultimately leading to the displacement or decline of native populations (Anton et al., 2019; Parker et al., 1999). Both natural dispersal and human‐mediated introductions facilitate the movement of marine organisms over long distances, enabling species to cross biogeographic boundaries and colonize new habitats (Creed et al., 2017; Joyeux et al., 2001). These processes can lead to substantial ecological restructuring, altering species composition, trophic interactions and ecosystem structure and function (Alidoost Salimi et al., 2021; Côté et al., 2013; Crooks, 2002; Green et al., 2012; Winnie Jr & Creel, 2017). While these changes are complex and beyond the reach of managers and stakeholders at local scales, such as those of Marine Protected Areas, the arrival, establishment and impacts of non‐native species must be understood to better address the issue at local scale by forecasting and mitigating their impacts. In this context, long‐term monitoring programs are critical research initiatives for detecting and understanding such ecological changes.

The Azores chromis *Chromis limbata* (Valenciennes, 1833) is a reef‐associated damselfish, native from the Macaronesian islands and the West African coast, from Senegal to Angola (Domingues et al., 2006; Wood, 1977), which has been sporadically recorded in the subtropical southwestern Atlantic since 2008 (Leite et al., 2009). The first records were made with sightings of two to five individuals on two coastal islands of Santa Catarina (27°S), southern Brazil, extending the known occurrences of the species over almost 6400 km (Leite et al., 2009). More recently, Anderson et al. (2017) documented the colonization progress of *C. limbata* off the coast of Santa Catarina and its potential impact on the populations of the brown chromis *Azurina multilineata* (Guichenot, 1853), a native species that likely shares a similar niche and a key planktivore on Brazilian reefs (Nunes et al., 2023). New occurrences of *C. limbata* were also recorded between Parcel de Torres (29°S), in the State of Rio Grande do Sul, and Cabras Island (23°S), off the coast of São Paulo State (Anderson et al., 2020), extending its known range both northward and southward from the first record in Brazilian waters. However, there is a gap of several hundred kilometres along the southeastern and southern coasts of Brazil where *C. limbata* has not yet been recorded.

The Alcatrazes Wildlife Refuge (AWR) (24°S), located 33 km off the southeastern Brazilian mainland, was established in 2016 and since then its reef fish communities have been monitored through multiple methodological approaches, including underwater visual censuses and Baited Remote Underwater Stereo‐Videos (Stereo‐BRUV; see Motta et al., 2021, 2024; Rolim et al., 2024). Here we used this exclusive dataset to document the recent arrival and increased abundance of *C. limbata* in the AWR, and to compare these patterns with the abundance of the local population of the native brown chromis, *Azurina multilineata*, discussing their potential ecological interactions. In addition, we provide evidence for the population increase of *C. limbata* in other coastal rocky reefs off São Paulo, where it was reported by Anderson et al. (2020) and Adelir‐Alves et al. (2018) (i.e. Laje de Santos Marine State Park and Queimada Grande Island). Finally, we contribute to the understanding of the non‐native occurrence of *C. limbata* on subtropical Brazilian reefs by recording a new occurrence at Parcel dos Reis, a submerged reef located between the coastal islands mentioned above.

The Alcatrazes Archipelago encompasses two no‐take MPAs: the Tupinambás Ecological Station (TES‐IUCN Category Ia), created in 1987, and the Alcatrazes Archipelago Wildlife Refuge (AWR‐IUCN Category III), established in 2016. These two MPAs cover a total area of 674.09 km^2^, including islands, islets and submerged rocky reefs up to 45 m deep (Motta et al., 2021; Roos et al., 2024). The Laje de Santos Marine State Park (Laje MSP‐IUCN Category II) was established in 1993 and covers a no‐take area of 500 km^2^, including its buffer zone. Located 36 km off the central coast of São Paulo, it consists of an islet and several sparse rocky reefs up to 50 m deep (Luiz Jr et al., 2008). Located approximately 40 km to the south, the Parcel dos Reis is one of several scattered and submerged rocky reefs inserted into a multiple‐use MPA (EPA‐IUCN Category V) between QGI and Laje MSP, at depths ranging from 13 to 30 m. The QGI has a heterogeneous subtidal substrate of up to 40 m, including a rocky reef associated with a relict coral reef that is the southernmost occurrence of coral‐built structure known in the South Atlantic (Niz et al., 2023; Pereira‐Filho et al., 2019, 2021).

Fish assemblages were monitored twice a year (summer and winter) between 2015 and 2024 (except in 2021 due to COVID‐19 pandemic restrictions) at seven sites around the main island of the AWR. At each site, stationary underwater visual censuses (UVC) (Minte‐Vera et al., 2008) were conducted at depths between 2.5 and 26 m. A total of 2431 UVCs were performed (2015: 2 sites; 2016: 3 sites; 2017 to 2024: 7 sites; Supporting Information Table S1). An average of 270 censuses were carried out per year [minimum 59, maximum 403, standard deviation (SD) 115.7], with an average of 45 per site (minimum 25, maximum 68, SD 12.8). In addition, stereo‐BRUV samplings were conducted from 2022 to 2024 at the same seven sites and one additional site, Ilha do Farol (Figure 1), approximately 700 m west of the sheltered side of the main island. Two stereo‐BRUV deployments were conducted annually at each site (i.e. summer and winter), except at the Ilha do Farol, where four deployments were conducted. Each deployment remained on the seabed recording for 60 min. A total of 18 h of stereo‐video footage were analysed per year across eight sites, resulting in 48 h analysed footage across all years. At QGI, Parcel dos Reis and Laje MSP, *C. limbata* was recorded by stereo‐BRUV (see Gragnolati et al., 2024) and recreational SCUBA videos.

**FIGURE 1:**
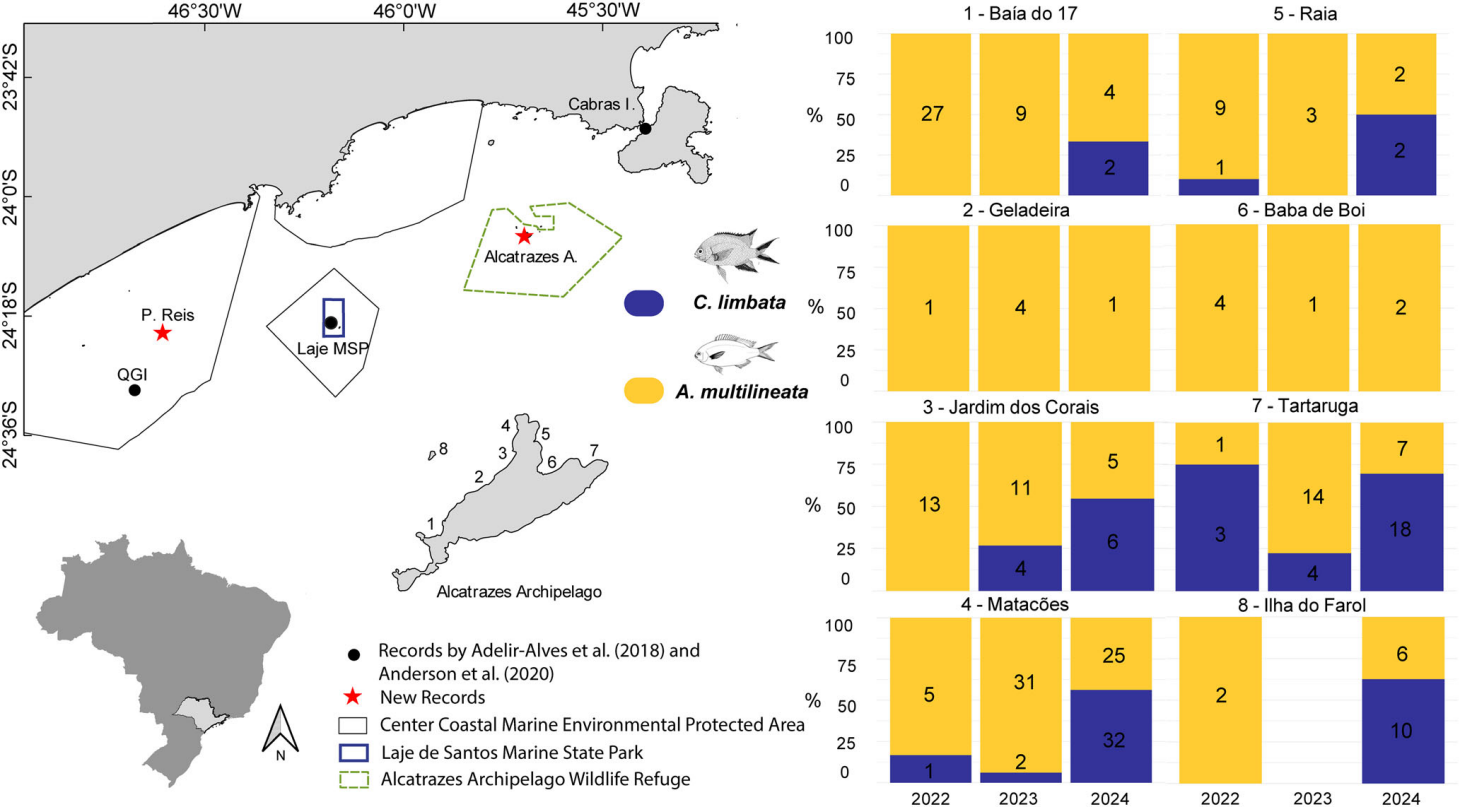
Map of the study area (on the left) showing the marine protected areas where *Chromis limbata* has been recorded on the coast of São Paulo, southeastern Brazil. Black circles represent previous records (Adelir‐Alves et al., 2018; Anderson et al., 2020) and red stars represent new records from the present study. Bar charts demonstrate the proportion (%) of individuals of *C. limbata* (blue) and *Azurina multilineata* (yellow) recorded at the different sites from 2022 to 2024. Numbers indicate total abundance of each species per site and year, based on combined data from UVC and stereo‐BRUVs.

On Queimada Grande Island we reported additional occurrences of *C. limbata* (Figure 2e,f). In December 2020, a vagrant individual was recorded by stereo‐BRUV at 12 m depth at Bananal site (24°29′17.30″S, 46°40′37.38″W). More recently, in February 2024, groups of three, six and eight individuals were observed at depth of 10 m at Naufragio site (24°28′57.66″S, 46°40′42.12″W). In June 2024, groups of 2–24 individuals were recorded at 12 m depth at the same site, all recorded by recreational SCUBA diving.

**FIGURE 2:**
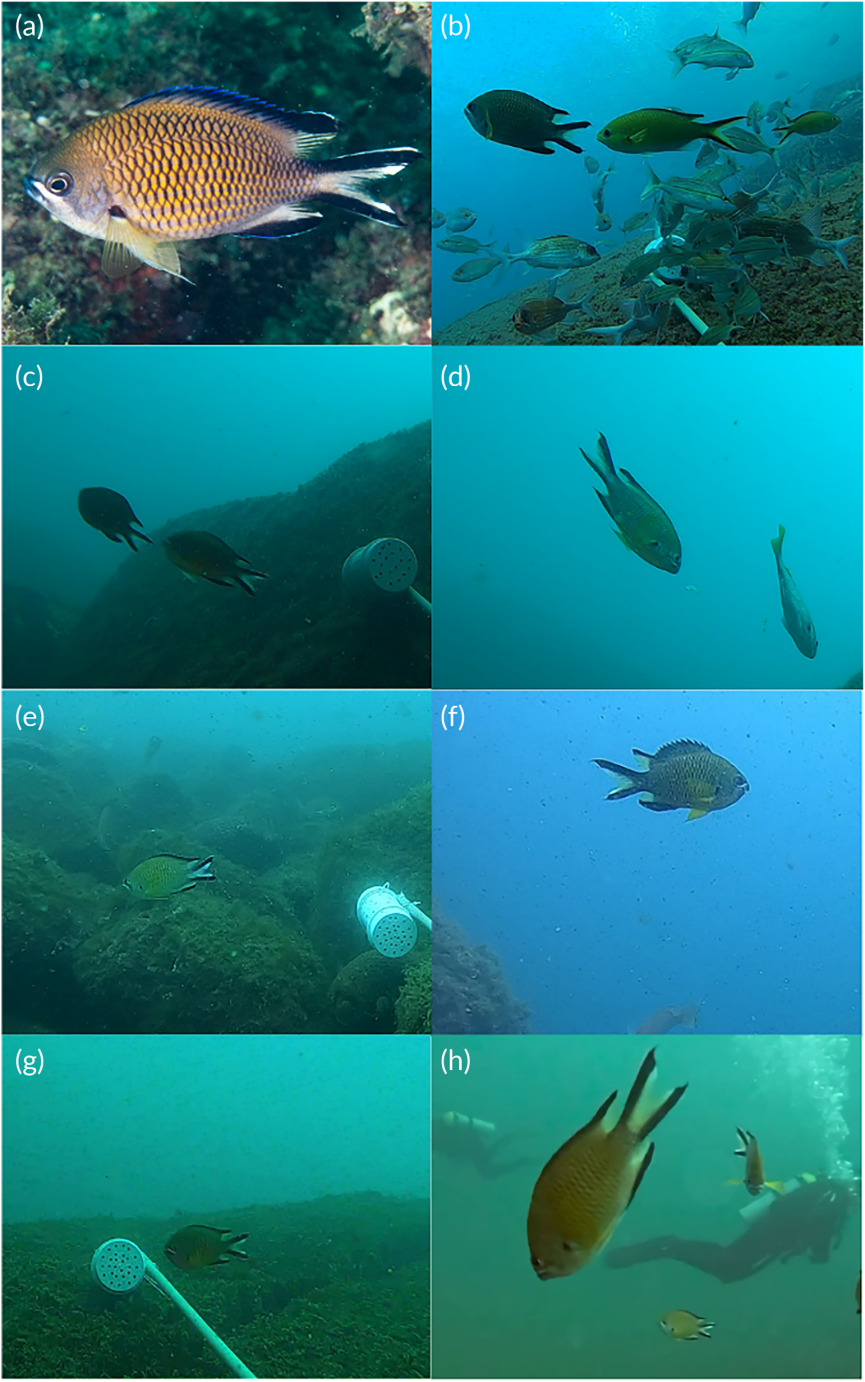
Photographs of *Chromis limbata* from different locations of the São Paulo State, southeast Brazil: (a) Alcatrazes Archipelago (image by L. Francini); (b) Alcatrazes Archipelago, with *Azurina multilineata*; (c and d) Parcel dos Reis; (e and f) Queimada Grande Island; (g and h) Laje de Santos.

A new record for the species was documented in 2021 at Parcel dos Reis (24°20′35.50″S, 46°36′24.12″W), a rocky reef located 18 km north of QGI along the coast and approximately 25 km offshore. At a depth of 17 m, two individuals (stereo‐BRUV: 9 cm TL) were recorded at this site (Figures 1 and 2c,d).

We also documented the records of *C. limbata* on Laje MSP, about 42 km from Parcel dos Reis, highlighting the establishment of the species on other islands and reefs in the subtropical southwestern Atlantic. In June 2024, a vagrant individual was recorded at the Portinho site (24°19′8.60″S, 46°10′54.36″W) at 15 m depth. At the same site, in December 2024, a group of 31 individuals was sighted during recreational SCUBA diving and two other individuals (>10 cm TL) were recorded with BRUVs at 19 m depth (Figure 2g,h).

Regarding the AWR, the first record of *C. limbata* was made in December 2017, with five large individuals (UVC >10 cm TL) between 12 and 13.5 m depth at Geladeira site, on the east sheltered side of the island (Figure 1; Supporting Information Table S2). That year, *A. multilineata* was recorded at all sites (mean depth 8.3 m), representing 97.8% of the proportion between both species. In 2018 and 2019, none *C. limbata* was sighted, while *A. multilineata* was recorded at all sites: 87 individuals in 2018 (mean depth 6.6 ± 0.8 m, TL 12.4 ± 9.6 cm) and 120 in 2019 (mean depth 7.5 ± 2.2 m, TL 17.1 ± 14.8 cm), with site counts ranging from two to 33 individuals. In 2020, 34 specimens of both species were recorded, being a single large individual of *C. limbata* (UVC >10 cm TL) was again only recorded at Geladeira site, at 9.8 m depth (2.9% of the total), while *A. multilineata* was recorded at all sites (one to 12 individuals per site, mean 4.7 ± 3.9, mean depth 8.0 ± 0.8 m). In 2022, increased efforts to employ stereo‐BRUVs led to the observation of *C. limbata* individuals at three additional sites. One individual (UVC 2–10 cm TL) at a depth of 11 m at Matacões, the northwesternmost point of the main island (~800 m from Geladeira). At site Raia, further northeast of the island (~1300 m from Geladeira), a single individual (UVC 2–10 cm TL) was seen at 12.6 m depth. Finally, three larger individuals (BRUV TL 10.6 ± 0.1 cm) were recorded at 17 m depth in Tartaruga, the deepest and most northeastern site of the island (~ 900 m from Raia). This was the first site to show a higher abundance of *C. limbata* compared to *A. multilineata* (75% of the individuals). Even with the increase in the number of sites with *C. limbata* records, *A. multilineata* dominated the total proportion of individuals with 92.5% of the 67 individuals (mean depth 8.9 ± 4.8 m). In 2023, there was an increase in both the number of individuals sighted and the total proportion, 10 individuals of *C. limbata* (12%) and 73 of *A. multilineata* (88%). The Azores chromis was sighted in three sites, with Jardim dos Corais being a new site with no previous records, increasing its occurrence to six of the eight monitored sites. Four individuals were seen at Jardim dos Corais (UVC 3, BRUV 1, TL 6.3 ± 0.6 cm, depth 6–10.6 m), two individuals at Matacões (UVC 2–10 cm TL, depth 10–17 m) and four individuals at Tartaruga (UVC 1, BRUV 3, TL 10.3 ± 3.6 cm, depth 10–17 m), while *A. multilineata* was recorded at mean water depth of 7.9 ± 3.7 m. The year 2024 showed the greatest population expansion and dominance in abundance of *C. limbata* compared to *A. multilineata*. A total of 122 individuals were sighted, 70 *C. limbata* (57.4%) and 52 *A. multilineata* (42.6%), observed at mean depths of 11.4 ± 3.6 and 8.2 ± 3.9 m, respectively. In this last year, the territorial expansion and the dominance of the non‐native species in relation to the native one became evident, with the dominance occurring in four sites (Jardim dos Corais, Matacões, Tartaruga and Ilha do Farol) of the Alcatrazes Archipelago. The same number of individuals of both species was observed at Raia, while three sites showed lower density of *C. limbata* compared to the native *A. multilineata*. However, during the last summer (2025) expedition to the Alcatrazes Archipelago, we recorded *C. limbata* at Baba de Boi and Geladeira. These were the first records of *C. limbata* at these sites, corroborating its continued territorial expansion on this island.

Here, we documented the establishment and population expansion of the non‐native species *Azores chromis (C. limbata)* along the coast of São Paulo state, including new records in areas where it had not been previously reported (Parcel dos Reis and the Alcatrazes Wildlife Refuge–AWR), as well as multiple individuals in areas where the species had been previously detected only as isolated occurrences (Queimada Grande Island–QGI and Laje de Santos Marine State Park–Laje MSP) (Adelir‐Alves et al., 2018; Anderson et al., 2020). For the AWR, we also provided evidence of a spatiotemporal increase in *C. limbata* abundance.

Regarding the *C. limbata* population expansion, the central coast of São Paulo is characterized by a diverse array of islands, rocky reefs and submerged reefs, with depths ranging from 10 to 40 m (Ângelo & Lino, 1989; Motta et al., 2021, 2022). This environmental feature creates a network of interconnected reef environments, which can be used as a stepping stone for Azores Chromis to sites further north, including Laje MSP and AWR. Additionally, this coastal region is characterized by factors such as the seasonal intrusion of the South Atlantic Central Water (SACW) and the formation of a thermocline during the summer months (Carvalho et al., 2025; Castro Filho & Miranda, 1998). These factors could provide favourable conditions for the *C. limbata*, a cold‐affinity species (Anderson et al., 2020). In this context, our results corroborate the species' capacity to thrive in the local environment, thereby indicating the likelihood of its population stabilizing in the forthcoming years. Moreover, our findings support the prognosis made by Anderson et al. (2020) that a healthy, increasing population has the potential to exhibit a higher density than *A*. *multilineata*.

At AWR, *C. limbata* has shown a population increase coinciding with the decline of *A. multilineata* in the last 3 years. The same pattern was also recorded years earlier further south by Anderson et al. (2017). Although we do not have systematic data on fish assemblages in QGI and Laje MSP, we have observed a notable population prominence of *C. limbata* compared to *A. multilineata* based on diving surveys and BRUV deployments conducted since 2015 at these sites, as part of broader research efforts by our group in the region.

Although *C. limbata* and *C. multilineata* share a similar zooplanktivorous diet (Froese & Pauly, 2025), the elevated plankton availability in the South Atlantic, primarily sustained by coastal upwelling processes (Castro Filho & Miranda, 1998; Pires‐Vanin et al., 1993), suggests that food limitation is unlikely to be a major driver of interspecific interactions (Anderson et al., 2015, 2017; Elleouet et al., 2014).

Alternatively, competition for non‐trophic resources, particularly shelter and nesting sites, may play a more relevant role. This competition could be further intensified by the presence of other highly abundant Pomacentridae species in the region, notably *Stegastes fuscus* (Cuvier, 1830) and *Abudefduf saxatilis* (Linnaeus 1758), both of which display pronounced territoriality and aggressive behaviour during reproductive periods. Such interactions may enhance competitive asymmetries for access to shelter and nesting substrates, potentially favouring the reproductive success of certain species while constraining others at meso‐ to large spatial scales (Anderson et al., 2017, 2020; Green et al., 2012). However, the extent to which these processes drive the observed patterns remains uncertain, underscoring the need for further investigation into the underlying ecological mechanisms.

These results are important as they highlight changes in the taxonomic structure of the reef fish community, involving both a native and a non‐native species. However, further studies, such as stable isotope analyses to investigate trophic position and habitat use, along with experimental approaches to assess competition, are needed to enhance our understanding of the ecological niches and environmental requirements of these species. Such studies should also include systematic reef fish monitoring to support a more comprehensive evaluation of the underlying causes and long‐term impacts of these changes, as well as the implementation of control measures if necessary.

## AUTHOR CONTRIBUTIONS

**R.R.M.:** Conceptualization, data collection, formal analysis, writing – original draft, writing‐review, and edition. **A.C.S.A., C.R.P.** and **F.A.R.:** Conceptualization, data collection and writing – review and editing. **G.H.P.F.:** Writing – review and editing. **F.S.M.:** Conceptualization, data collection, writing – review and editing, supervision, project administration, funding acquisition. All authors read and approved the final version of the manuscript.

## FUNDING INFORMATION

Financial support was provided by FAPESP (Process Number 2019/19423–5 and 2023/11845–3), Instituto Linha D'água and Petrobras (Tripartite Agreement FapUnifesp‐Unifesp–Petrobras #23089102938/2019–54–Mar de Alcatrazes Project). RR Munhoz thanks to FAPESP for the Masters scholarship (Process Number 2024/12826–5). G.H. Pereira‐Filho and F.S. Motta acknowledge individual grants from the Brazilian Research Council (CNPq).

## Supporting information


**TABLE S1.** Sampling effort UVS/BRUV.

**TABLE S2.** Species records.
